# Interleukin 18 and IL-18 BP response to Sars-CoV-2 virus infection

**DOI:** 10.1007/s10238-022-00943-9

**Published:** 2022-11-16

**Authors:** Luca Marino, Anna Criniti, Sofia Guida, Tommaso Bucci, Laura Ballesio, Marianna Suppa, Gioacchino Galardo, Alessandra Vacca, Maria Santulli, Antonio Angeloni, Carla Lubrano, Orietta Gandini

**Affiliations:** 1 Emergency Medicine Unit, Department of Emergency‐Acceptance, Critical Areas and Trauma, Policlinico “Umberto I”, 00161 Rome, Italy; 2 Department of Mechanical and Aerospace Engineering, “Sapienza” University of Rome, 00168 Rome, Italy; 3 Department of Experimental Medicine, Sapienza University of Rome, Rome, Italy; 4 Department of General Surgery and Surgical Specialties “Paride Stefanini”, Sapienza University of Rome, Rome, Italy; 5 Department of Radiology, Anatomo-Pathology and Oncology, Sapienza University of Rome, Rome, Italy; 6 Department of Molecular Medicine, Sapienza University of Rome, Rome, Italy

**Keywords:** Interleukin-18, Interleukin-18 binding protein, INF-γ, Sars-CoV-2, P/F ratio, Severe acute respiratory syndrome, Pneumonia, Tomography X-ray computed

## Abstract

The immune response to the SARS-CoV-2 infection is crucial to the patient outcome. IL-18 is involved in the lymphocyte response to the disease and it is well established its important role in the complex developing of the host response to viral infection. This study aims at the analysis of the concentrations of IL-18, IL-18BP, INF-γ at the onset of the SARS-CoV-2 infection. The serum levels of measured interleukins were obtained through enzyme-linked immunosorbent assay. Furthermore, the free fraction of IL-18 was numerically evaluated. The enrolled patients were divided in two severity groups according to a threshold value of 300 for the ratio of arterial partial pressure of oxygen and fraction of inspired oxygen fraction and according to the parenchymal involvement as evaluated by computerized tomography at the admittance. In the group of patients with a more severe disease, a significant increase of the IL-18, INF-γ and IL-18BP levels have been observed, whereas the free IL-18 component values were almost constant. The results confirm that, at the onset of the disease, the host response keep the inflammatory cytokines in an equilibrium and support the hypothesis to adopt the IL-18BP modulation as a possible and effective therapeutic approach.

## Introduction

The recent pandemic of COVID-19, now moving to an endemic condition, put a significant emphasis on the immune response of the host to the virus SARS-CoV-2 infection [1]. Terms like “cytokines storm” became sadly common in the critical phase of the disease and it is now well known that the main clinical condition associated is an acute respiratory distress syndrome (ARDS), a crucial state for the unfavorable prognostic of patients [2].

Cytokines storm syndromes encompassed a set of clinical conditions mainly related to infection disease, in particular of viral nature [3], defined as a cascade of exaggerated events that lead to a hyperinflammation environment which might ultimately result in multiple organ failure [4]. Cytokines storm was deeply studied in the past by several authors for macrophage activation syndrome (MAS), adult-onset Still's disease (AOSD), catastrophic anti-phospholipid syndrome (CAPS) and septic shock [5] and hyperferritinemic syndrome has been recognized as a consequence of hyperinflammation [6–9]. The severe form of COVID-19, which shares many clinical and laboratory characteristics with this syndrome, was recognized to have a time history characterized by three phases: an early response with mild constitutional symptoms and sometimes lymphopenia (phase I), a phase II with the onset of a peculiar viral pneumonia and typical ground glass opacities at chest computed tomography (CT) and, in several cases, a hypoxic condition (defined, i.e., by a P/F ratio PaO_2_/FiO_2_ < 300 mm Hg) [10]. At phase II hospitalization become necessary. A small subset of these patients proceeds into the severe phase III with a systemic hyperinflammation syndrome featured clinically by ARSD, disseminated intravascular coagulopathy (DIC), shock and heart failure [11]. The laboratory values revealed high values of inflammatory cytokines and biomarkers (IL-2, IL-6, IL-7, TNF-α, ESR, CRP, ferritin, and D-dimer) [5].

A possible classification of the main patterns of cytokines inflammatory mechanisms is based on the axes IL-6/CRP and IL18/Ferritin [12] as infection programs related to bacterial and viral infections. A clear dichotomy between the two routes is not well defined and it was proved that the plasma levels of both cytokines IL-6 and IL-18 are significantly associated with the disease severity of the SARS-CoV-2 infection [13, 14].

The interest for these cytokines as biomarkers for prognostic purpose is promising to evaluate the possible therapeutic strategy to modify the clinical course of the disease and to check the effectiveness of the patient response to the treatment.

IL-18 belongs to the pro-inflammatory IL-1 family of cytokines and its expression can be induced by several inflammatory stimuli. Several studies analyzed infections (particularly viral), metabolic and inflammatory diseases (adult-onset Still’s disease, systemic juvenile idiopathic arthritis, hemophagocytic lympho-histiocytosis/macrophage activation syndrome) where IL-18 is an important actor of the host response [15].

The biological action of IL-18 is strictly related to the protein IL-18BP which is constitutively secreted by mononuclear cells and displays a remarkably high affinity for IL-18 (400 pM) [16]. It can be found in the serum of healthy humans at a 20-fold molar excess compared to IL-18 [17]. Its action, as decoy receptor, negatively regulates the biological function of the IL-18 through a down-regulation of the autoimmune response of the host, to avoid a systemic damage. In fact, the expression of IL-18BP is related, in a feed-back loop, to the INF-γ which enhances its expression [18].

To note, the net biological effect of IL-18 is actually given by the fraction of IL-18 not bound to IL-18BP, the IL-18 free, and so dependent on balance between the concentrations of the two proteins. The levels of circulating IL-18, IL-18BP and the corresponding values of IL18 free were considered in several paper for different diseases in relation to the disease characteristics and severity [18, 19]. In general, high levels of IL-18BP are found in physiological conditions and, in the presence of an inflammatory disorders, a further increase has been observed [20, 21]. Though this effect is partially explained by the concomitant increase of IL-18 and its feed-back action through INF-γ, in some cases a significant dysregulation has been witnessed in the ratio IL-18/IL-18BP.

In patients with viral infections, high levels of IL‐18 free have been recognized [22] suggesting the important role of IL‐18 in the cytokine storm, along with IL‐1β and IL‐6, as in the case of COVID‐19. In particular, in viral diseases the imbalance of the IL18/IL-18BP ratio appears particularly significant for the pathophysiology of the abnormal response to the infection [23].

Particularly significant is the possible use of human recombinant IL-18 BP as a therapeutic approach to control the derangement of the immune response in the severe phase of the disease [24–26].

Aim of this paper is to evaluate the measured plasma levels of IL-18, IL18BPm INF-γ and the corresponding IL-18 free, in a set of patients affected by COVID-19 infection, at the admission in the Emergency Department.

## Materials and methods

A cohort of 85 COVID-19 patients, admitted to the Emergency Medicine Department of the Policlinico Umberto I hospital in Rome between March 2020 and June 2021 was selected to assess the cytokines profile at the admission time. The diagnosis of COVID-19 was confirmed by means of two positive polymerase chain reaction (PCR) tests carried out on nasopharyngeal swab samples.

A routine laboratory screening was carried out, including, in particular, the complete blood count (CBC), lactate dehydrogenase (LDH), C-reactive protein (CPR), ferritin, d-dimer, troponin T, pro-thrombin time (PT), activated partial thromboplastin time (aPTT), creatine phosphokinase (CPK), electrolytes, renal acute injury parameters and liver enzymes. An arterial blood gas analysis at the admittance was also executed and the corresponding PaO_2_/FiO_2_ ratio (P/F ratio) was calculated.

In addition to these parameters, all the patients executed a chest CT examination and a measurement of serum concentrations of IL-6, IL-18, IL-18BP, and INF-γ.

IL-6, IL-18,IL18BP and INF-γ were measured in the serum of the patients by means of ELISA assay produced by Thermo-Fischer (Human IL-6 ELISA kit with analytical sensitivity < 1.0 pg/mL, assay range 1–200 pg/mL, Human IL-18 ELISA Kit with analytical sensitivity 9.0 pg/mL, assay range 78–5,000 pg/mL and IL-18BP Human ELISA Kit with analytical sensitivity 20.0 pg/mL, assay range 24.18–26,000 pg/mL, Human INF-γ ELISA Kit with analytical sensitivity 0.16 ng/mL, assay range 0.16–40 ng/mL).

The values of the IL-18 free were calculated according to the action mass law. A 1:1 stoichiometry ratio for the complex IL-18/IL-18BP and a dissociation constant *K* = 0.4 nM were assumed. The molecular weight adopted for IL-18 and IL-18BP are 18.4 kDa and 17.6 kDa, respectively [17, 21].

The patients were stratified based on two possible severity scores.

A cut-off value of the first measured P/F = 300 mmHg ratio was considered as first score, while a second severity index (SI) was evaluated based on the lung involvement at the hospital admittance. The lung involvement, reported as percentage of parenchyma interested by the disease, was established through the analysis of the chest CT by expert radiologists following a standardized procedure [10, 27]. In particular, the SI was 0 or 1 according to a pulmonary parenchyma involvement threshold of 50%.

### Statistical analysis

Categorical variables were reported as frequency distribution, whereas continuous variables were described through median and interquartile range (IQR) or mean value and standard deviation.

A Mann–Whitney *U* test was adopted to compare the measured values between the groups and a *p* value < 0.05 was assumed to be statistically significant.

A Pearson correlation analysis with a two tails significance test was exploited for the most important variable and the receiver operating characteristic (ROC) curve with the area under the curve (AUC) was evaluated to test the predictive value against a worse outcome.

All the analyses were executed using the IBM software SPSS 25.

## Results

The mean age of the enrolled 85 patients is 56.7 ± 19 years and 53.6% were male. The two groups, divided according to the P/F = 300 value, show a moderate but significant, difference in the mean age, 52.7 ± 17 years (P/F > 300) vs 64.85 ± 18 years (P/F < 300) (*p* = 0.011).

The mean value of P/F for all the patients is 325 ± 102, but a meaningful difference is observed between the two P/F groups: 407 ± 64 (P/F > 300), 227 ± 59.7 (P/F < 300), *p* < 0.001.

As expected, the IL-6 is significantly different in the group of patients with P/F < 300 with respect to those with P/F > 300 and to complete sample In detail, IL-6 = 78.6 ± 54.7 pg/mL (P/F < 300), IL-6 = 17.8 ± 19.2 pg/mL (P/F > 300), *p* = 0.001.

The value of IL-18 does not change significantly between the three groups presents. The mean values are: IL-18 = 508 ± 299.5 pg/mL (all patients), IL-18 = 477 ± 354 pg/mL (P/F > 300), IL-18 = 583 ± 301 pg/mL (P/F < 300), *p* = 0.07.

The measured IL-18BP is 2716 ± 1536 pg/mL for the complete group, but a significant difference is recognized between the patients with P/F > 300 (IL-18BP = 2170 ± 1170 pg/mL) and the patients with P/F < 300 (IL-18BP = 3489 ± 1804 pg/mL), with *p* = 0.001.

The free component of IL-18 is almost constant for all the three different classifications. In detail, IL-18free = 371 ± 220 pg/mL (all patients), IL-18free = 371 ± 280 pg/mL (patients with P/F < 300), IL-18free = 393 ± 192 pg/mL (patients with P/F < 300), *p* = 0.24.

The results of measured INF-γ are particularly interesting. A significant increase of the concentrations of INF-γ is observed for the patients with P/F < 300 with respect to those with P/F > 300 and to the complete sample. The measured mean values are INF-γ  = 7.41 ± 3.6 ng/mL (patients with P/F < 300), INF-γ = 2,72 ± 3.29 ng/mL (patients with P/F > 300), *p* = 0.015, and INF-γ = 4.67 ± 3.59 ng/mL for all the patients.

The other routinely laboratory parameters CRP, ferritin and D-dimer assume significant larger values in the more severe P/F < 300 group, as expected. Values of blood cells counts do not display any meaningful differences between the groups and fall in the normal range.

The main comorbidities recorded are: Arterial hypertension (31%), chronic obstructive pulmonary disease (2.4%), diabetes (8.3%), atrial fibrillation (2.3%), neoplastic disease (2.4%), and chronic heart failure (6%). Noteworthy differences are observed between the two groups for patients with chronic obstructive pulmonary disease, neoplastic disease, and chronic heart failure.

The mean values and the standard deviations of all the measured variables with the corresponding statistical significance are reported in Table 1.

**Table 1:** Characteristics of the patients. Mean values and standard deviations for the complete groups and for the two sub-groups according to threshold value P/F = 300

	All patients (*N* = 85)	P/F > 300 (*N* = 45)	P/F < 300 (*N* = 40)	*p* value
Age (years)	56.7 (19)	52.7 (17)	64.5 (18)	0.011
P/F (admittance value)	325 (102)	407 (66.4)	227 (59.7)	< 0.001
IL-6 (n.r. 0–46 pg/mL)	47.7 (109)	17.8 (19.2)	78.6 (54.7)	0.001
IL-18 (n.r. 70–490 pg/mL)	508.8 (299.5)	477 (354)	583 (301)	0.07
IL-18BP (n.r. 2000–3000 pg/mL)	2716 (1536)	2157 (1170)	3489 (1804)	0.001
IL-18free (pg/mL)	371 (220)	371 (280)	393 (192)	0.246
INF-γ (ng/mL)	4.67 (3.59)	2.73 (3.29)	7.41 (3.6)	0.015
CRP (n.r. < 0.5 mg/dL)	5.57 (6.86)	3.18 (6.9)	8.15 (7.03)	0.006
Ferritin (n.r. 30–400 ng/mL)	863 (1032)	497 (473)	1218 (1434)	0.006
D-Dimer n.r. < 500 ng/mL)	886 (866)	555.6 (348)	1422 (1108)	0.001
Creatinine (n.r. 0.1–0.9 mg/dL)	0.85 (0.23)	0.85 (0.21)	0.86 (0.22)	0.8
Red blood cells (n.r. 3.5–5.1 × 10^6^/μL)	4.76 (0.59)	4.18 (0.55)	4.63 (0.70)	0.236
White blood cells (n.r. 4–10 × 10^3^/μL)	13.5 (1.5)	13.5 (1.3)	13.2 (1.8)	0.581
Neutrophils (n.r. 2.2–6.6 × 10^3^/μL)	5.09 (3.2)	5.06 (2.8)	4.95 (2.9)	0.877
Lymphocytes (n.r. 1–3.2 × 10^3^/μL)	1.01 (0.5)	0.95 (1.1)	1.01 (0.5)	0.290
Platelets (n.r. 50–450 × 10^3^/μL)	205 (85)	208 (70)	218 (114)	0.672
Comorbidities				
Arterial Hypertension (%)	31	11	20	0.019
Chronic obstructive pulmonary disease (%)	2.4	0	2.4	n.a
Diabetes (%)	8.3	2.2	6.1	0.147
Atrial Fibrillation (%)	2.3	1.1	1.2	0.273
Neoplastic disease (%)	2.4	0	2.4	n.a
Chronic heart failure (%)	6	2.3	3.7	0.011

The values of the measured cytokines and inflammatory indexes, according to the severity index SI, based on lung parenchymal involvement, are reported in Table 2. This different approach to evaluate the severity of the disease in the patients, presents analogous results compared to the P/F parameter. In particular, IL-6, IL-18BP and INF-γ display a significant increase in the group corresponding to a higher radiological severity (CT with > 50% of parenchymal involvement). In detail, IL-6 = 66 ± 52.6 pg/mL (SI = 1), 36.3 ± 42 pg/mL (SI = 0), *p* = 0.005. IL-18BP = 3325 ± 1288 pg/mL (SI = 1), 2360 ± 1077 pg/mL (SI = 0), *p* = 0.005. INF-g = 7.01 ± 4.1 pg/mL (SI = 1), 3.01 ± 2.71 pg/mL (SI = 0), *p* = 0.025. The values of CRP, Ferritin and D-Dimer are larger in the more severe group SI = 1, but only Ferritin present a difference statistically significant (*p* = 0.014).

**Table 2:** Characteristics of the patients according to severity index SI. SI = 1 corresponds to lung parenchymal involvement > 50% as evaluated on chest CT

	SI = 0 (*N* = 55)	SI = 1 (*N* = 35)	*p* value
IL-6 (n.r. 0–46 pg/mL)	36.3 (42)	66.9 (52.6)	0.005
IL-18 (n.r. 70–490 pg/mL)	491 (301)	538 (299)	0.485
IL-18BP (n.r. 2000–3000 pg/mL)	2360 (1077)	3325 (1288)	0.005
IL-18free (pg/mL)	370 (232)	371 (201)	0.995
INF-γ (ng/mL)	3.01 (2.71)	7.01 (4.1)	0.025
CRP (n.r. < 0.5 mg/dL)	4.87 (6.53)	6.71 (7.3)	0.242
Ferritin (n.r. 30–400 ng/mL)	653 (656)	1222 (1410)	0.014
D-Dimer (n.r. < 500 ng/mL)	760 (716)	1111 (1061)	0.080

Table 3 shows the Pearson correlation coefficients with the statistical significance for the measured concentration of IL-6, IL-18, IL-18BP, IL-18 free, CPR, Ferritin and D-dimer.

**Table 3:** Pearson correlation coefficients and p-value for the measured concentration of IL-6, IL-18, IL-18BP, IL-18 free, INF-γ, Ferritin, CPR, D-dimer

Pearson correlation coefficient r	IL-6	IL-18	IL-18BP	IL-18 free	INF-γ	Ferritin	CRP	D-Dim
IL-6		0.229 (*p* = 0.038)	0.412 (*p* < 0.001)	0.093 (*p* = 405)	0.022 (*p* < 0.001)	0.435 (*p* < 0.001)	0.559 (*p* < 0.001)	0.459 (*p* < 0.001)
IL-18			0.307 (*p* = 0.005)	0.996 (*p* < 0.001)	0.16 (*p* = 0.15)	0.207 (*p* = 0.059)	0.133 (*p* = 0.233)	0.129 (*p* = 250)
IL-18BP				0.307 (*p* = 0.005)	0.14 (*p* = 0.27)	0.420 (*p* < 0.001)	0.410 (*p* < 0.001)	0.271 (*p* = 0.015)
IL18 free					0.037 (*p* = 0.54)	0.091 (*p* = 0.411)	0.025 (*p* = 0.827)	0.071 (*p* = 0.513)
INF-γ						0.411 (*p* < 0.001)	0.18 (*p* = 0.09)	0.07 (*p* = 0.12)
Ferritin							0.405 (*p* < 0.001)	0.23 (*p* = 0.02)
CRP								0.204 (*p* = 0.005)
D-Dim								

IL-6 has a significant positive correlation with IL18-BP (*r* = 0.412, *p* < 0.001), CPR (*r* = 0.559, *p* < 0.001), Ferritin (*r* = 0.435, *p* < 0.001) and D-dimer (*r* = 0.459, *p* < 0.001). IL-18 with IL18-BP (0.307, *p* = 0.005) and IL-18 free (*r* = 0.996, *p* < 0.001). IL18BP with IL18 free (*r* = 0.307, *p* = 0.005), CRP (*r* = 0.420, *p* < 0.001), Ferritin (*r* = 0.410, *p* < 0.001). No significant correlations are observed for INF-γ, expect with Ferritin (*r* = 0.411, *p* < 0.001).

**Fig. 1:**
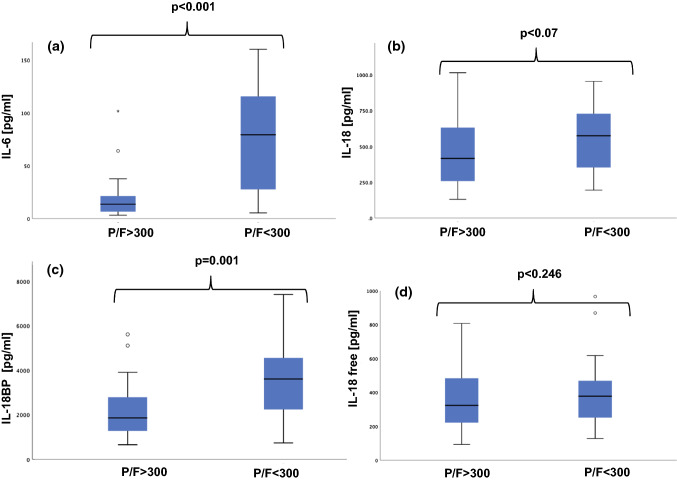
Boxplots relative to the measured cytokines. Median and interquartile ranges. Panel **a** IL-6. P/F > 300: 13.6 (6.4–22.4) pg/ml, P/F < 300: 79.3 (26.1–120) pg/ml; Panel **b** IL-18. P/F > 300: 416 (254–632) pg/ml, P/F < 300: 574 (344–742) pg/ml; Panel **c** IL-18BP. P/F > 300: 1857 (1271–2792) pg/ml, P/F < 300: 3607 (2172–4555) pg/ml; Panel **d** IL-18 free. P/F > 300: 322 (219.5–484) pg/ml, P/F < 300: 377 (247–477) pg/ml

Figure 1 shows the boxplots relative to IL-6, IL-18, IL-18BP, IL-18 free for P/F > 300 versus P/F < 300. Median levels are IL-6: 13.6 and 79.3 pg/ml (interquartile ranges, 6.4 to 22.4 and 26.1 to 120.0 pg/ml), IL-18: 416 and 574 pg/ml (interquartile ranges, 254 to 632 and 344 to 742 pg/ml), IL-18 BP: 1857 and 3607 pg/ml (interquartile ranges, 1271 to 2792 and 2172 to 4555 pg/ml), IL-18 free: 322 and 377 pg/ml (interquartile ranges, 219.5 to 484 and 247 to 477 pg/ml).

**Fig. 2:**
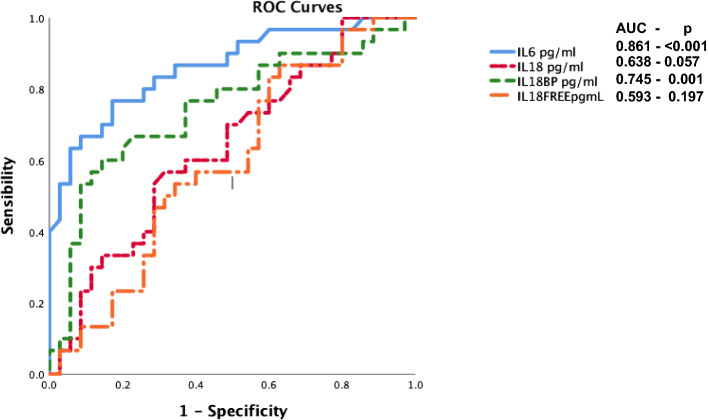
Receiver operating curves (ROC) and area under curve (AUC,) for IL-6, IL-18, IL-18BP, IL-18 free, for the P/F < 300 value

Figure 2 reports the receiver operator curve (ROC) of IL-6, IL-18, IL-18BP, IL-18 free, for the P/F = 300 value. IL-6 and IL-18BP have significant values of area under the curve AUC. In particular AUC = 0.861 (IL-6, *p* < 0.001), AUC = 0.745 (IL-18BP, *p* < 0.001).

**Fig. 3:**
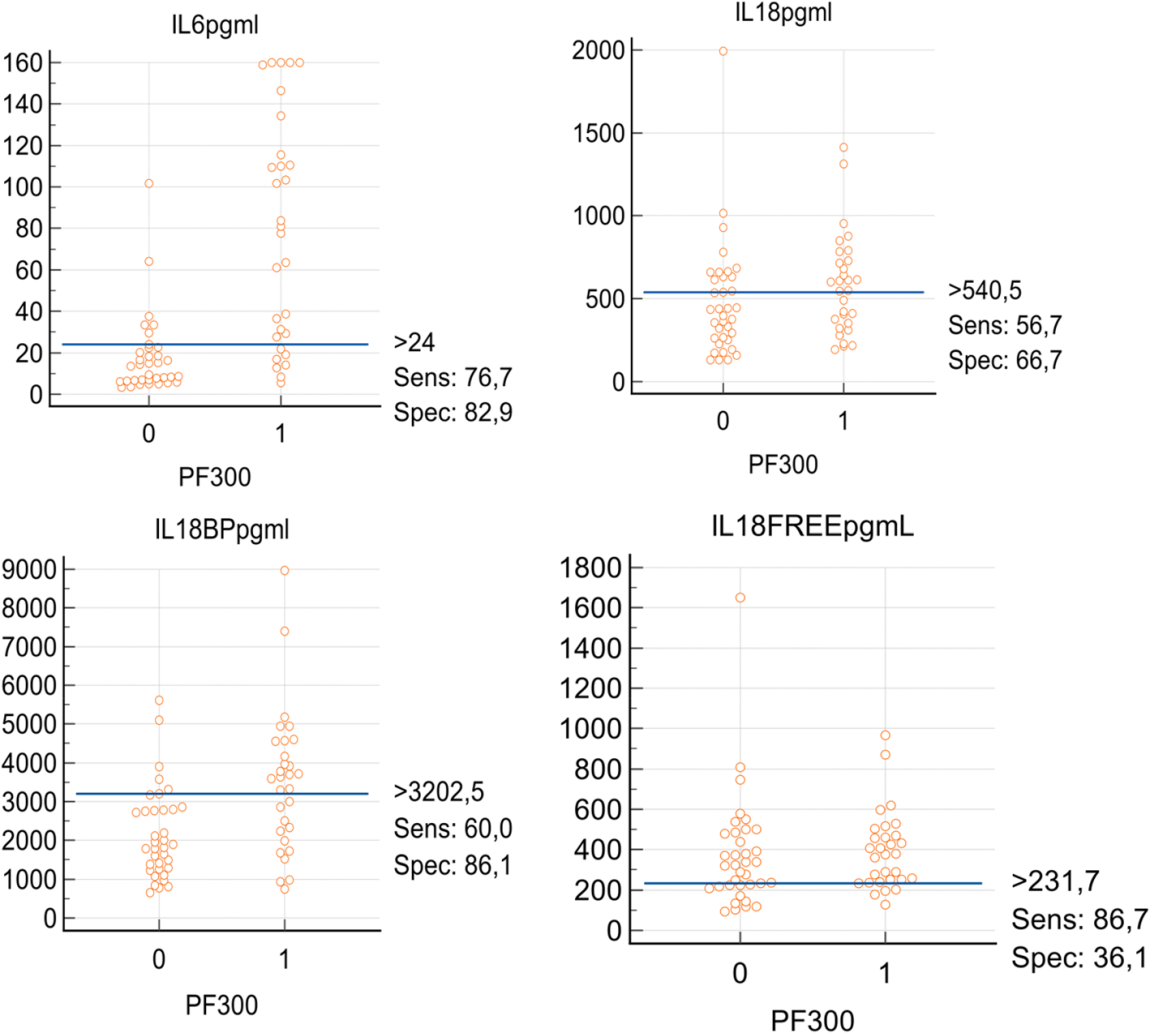
Interactive dot diagrams for IL-6, IL-18, IL-18BP and IL-18 free, for P/F > 300 (label 0) and P/F < 300 (label 1). The Youden index corresponds to the ROC curve analysis

Figure 3 shows the interactive dot diagrams for IL-6, IL-18, IL-18BP and IL-18 free, for P/F > 300 (label 0) and P/F < 300 (label 1). The Youden index corresponding to the ROC curve analysis is also reported.

## Discussion

The present study emphases the significant role of IL-18 in disease where immune system plays a crucial function. The analysis confirms the previously data concerning the important involvement of IL-6 in the inflammatory response to a virus infection but give also new insights on the mechanisms governing the IL-18 production and regulation in the case of human Sars-CoV2 infection.

The severity degree is well discriminated by the serum concentrations of IL-6, IL-18 and, in particular, IL-18BP. The results are independent on the particular index adopted to classify the disease severity and for both scores considered (P/F < 300 and percentage of lung parenchyma involvement) the concentrations of IL-18BP are higher when the pathology is more severe. The feedback mechanism related to INF-γ as an inducer of IL-18BP expression is confirmed by the higher values of the INF-γ in the more severe groups and the action of IL-18BP as a decoy receptor to dampen the IL-18 signal is proved by the almost constant concentrations of the biological active fraction IL-18 free.

Significant correlations were found between the inflammatory indexes (CRP, ferritin) and IL-6 and IL-18BP. IL-18 and IL-18BP showed good Pearson correlation coefficients with the free component of the IL-18 (*r* = 0.996 and *r* = 0.307, respectively) suggesting a strong regulation of the production of IL-18, IL-18BP and IL-18 free.

The receiver operator curve (ROC) shows the good prediction performance of IL-6 and IL-18BP for P/F < 300 (AUC 0.861 and 0.745, respectively, with significant p-values). Less predicting are the ROC curves for IL-18 and the IL-18 free (AUC 0.638 and 0.593 with p-values 0.057 and 0.197, respectively), in agreement to the “flat” response of IL-18-free in the initial phase of the disease.

From these preliminary results, we can conclude that IL-18BP plays a significant role in the modulation of immune response to IL-18, contributing to keep the biological active form, IL-18 free, to almost constant value, regardless of the severity of the disease.

This issue is particularly significant in view of the dynamic feature of the immune response that could shifts from protective to damaging in later stages. The timing of administration of a possible treatment, such as human recombinant IL-18BP, is still on debate. An early treatment could switch off the virus clearance by the immune system of the host, while a late intervention could result useless in controlling the cytokine mediated injury.

In conclusion, our study suggests that IL-18 and, maybe more important, IL-18BP are candidate to be adopted as severity prediction biomarkers and possible therapeutic targets.

It is worthwhile to note that the evolution of the immune response should be addressed with a follow up to track the equilibrium between IL-18, INF-γ and IL-BP and the possible transition to an uncontrolled inflammatory response of the host, typical of the phase III with ARSD. This study has the limit of a small sample size that could affect the statistical significance and further investigations, on larger sample size and at different time instants, are necessary to obtain a more complete description of the role of the ratio IL18/IL-18BP and role of INF-γ as a function of the disease severity degree.
